# HazAPP: Study protocol of a randomized controlled trial to enhance parental instruction of teens’ hazard anticipation skill during the learner phase of licensure

**DOI:** 10.1186/s40621-026-00695-0

**Published:** 2026-09-15

**Authors:** Elizabeth E. O’Neal, Ella Mucciolo, Giuliana Rokke-Smith, Brynn Swanson

**Affiliations:** The University of Iowa, Iowa City, IA USA

## Abstract

**Background:**

Despite very low crash rates during the learner phase of supervised licensure, teen driver crash rates immediately spike at the onset of independent licensure. A large percentage of driving errors among newly licensed teens is attributed to a failure to recognize roadway hazards. Parents serve as the primary instructor of teens’ driving under the learner phase of licensure, yet existing literature indicates that parents do not regularly engage in instruction on how to anticipate and respond to potential roadway hazards. The goal of the current randomized control trial, as reported in this study protocol paper, was to evaluate a web-based, parent-focused teen driving intervention, the Hazard Anticipation Program for Parents (HazAPP), to improve parental instruction of teens’ ability to anticipation hazards.

**Methods:**

Teen drivers in the learner phase of licensure (ages 14 to 17) and their parents were recruited via email listservs, postings and flyers to area schools, social media postings, and word of mouth. Pending eligibility screening and consent, dyads were randomly assigned to either the intervention or control arm of the study. Parents in the intervention arm of the study engaged in an online training program that sought to help parents better communicate their expert hazard anticipation knowledge to their teens. The training was two-fold: parents engaged in a web-based program that provided insights on how to best communicate with their teen when encountering hazards on the roadway, followed by a guided joint task that allowed parents to practice these skills with their teen in a safe environment by watching videos of hazards unfold. The study took place over two study visits approximately 4 weeks apart. At Visit 1, parents and teen completed questionnaires and engaged in an acclimation drive to ensure dyads would not experience motion sickness in simulated driving. All participants were then given a dashcam to record four naturalistic driving sessions before returning for Visit 2 where participants completed a study drive with 17 embedded hazards. Driving sessions (simulated and naturalistic) will be coded for content to determine intervention efficacy for improving parental instruction. Primary outcomes will include parental use of functional driving instructions, hazard identification, hazard anticipation communications, if these communications occurred before hazards become critical and if they contain elements of motivational interviewing, which were modeled in the web-based program. Secondary outcomes of teens’ driving performance will include hard braking events and lane changes in response to hazards and parental instructions.

**Trial registration:**

This study (NCT06611241) was registered on Clinicaltrials.gov on September 2, 2024 (https//clinicaltrials.gov/study/NCT06611241) and last updated on February 9, 2026. This protocol was developed using the SPIRIT guidelines and checklist. The version reported here is the first protocol version that was submitted on September 2, 2024.

**Supplementary Information:**

The online version contains supplementary material available at https://doi.org/10.1186/s40621-026-00695-0.

## Introduction

Teens experience the highest crash rates immediately upon beginning to drive independently [1–3] and are three times more likely to be involved in a fatal crash than adults (IIHS [4]), . A key contributor to elevated crash rates among novice teen drivers is an underdeveloped ability to identify potential hazards in the driving environment. Research has shown that over 45% of novice driver crashes are due to failure to anticipate and respond to a hazard [5]. Experienced drivers become adept at anticipating potential hazards, defined as emerging threats that may evolve into a crash, such as an upcoming lane closure or a child running into the street. Overall, teen crashes drop by 40% after 18 months of driving [1], indicating that novice drivers are learning to anticipate hazards as they gain independent, on-road driving experience.

Parental instruction is clearly a critical component of the supervised driving period. However, many experts in the field agree that more work is needed to understand how to support parents’ efforts to teach teens to drive [6], as relatively little is known about this process [7]. The few existing studies indicate that parents are not skilled in instructing their teens. For example, naturalistic studies examining parental driving instruction during supervised driving showed that parents primarily focus on functional driving instruction (relating to the immediate driving environment) such as vehicle handling and navigation [8–10]. Ehsani and colleagues [8] also found that functional instruction (e.g., vehicle handling, navigation) was highly correlated with driving errors, indicating that this form of instruction follows rather than precedes errors. Together, this demonstrates a need for a parent-focused intervention that trains parents to better prepare their teens to anticipate and respond to potential roadway hazards before teens begin to drive without supervision. The current manuscript presents the study protocol used to evaluate the feasibility of such an intervention.

### Objectives

The objective of the parallel randomized trial (ClinicalTrials.gov: NCT06611241) is to evaluate the feasibility of a parent-focused intervention, the Hazard Anticipation Program for Parents (HazAPP). HazAPP seeks to improve parental instruction of novice teen drivers’ hazard anticipation skills. We used driving simulation to test the feasibility of the parent-focused intervention by comparing parent instruction between dyads whose parents were randomly assigned to the intervention compared to those that were randomly assigned to the control condition. We hypothesize that parents in the intervention arm of the study will exhibit increased use of hazard anticipation instruction and will be more likely to do so before a hazard becomes critical.

## Methods

### Study design

The HazAPP study used a parallel, two-arm randomized controlled design to test whether a parent-focused, web-based intervention improved parent-teen communication about hazard anticipation during supervised driving. Parent-child dyads took part in two in-person visits over the course of approximately four weeks. This interval was chosen to provide parents with ample time to complete the training program and all four naturalistic study drives, could accommodate all drivers in the learner phase of licensure, and also reduced the likelihood of study attrition. The first visit included obtaining consent from parents and teens, parent and teen survey completion, and a brief acclimation drive in a medium-fidelity driving simulator with a motion base and a 135º field of view, the NADS-2. The acclimation drive lasted approximately eight minutes. The purpose of this acclimation drive was to ensure that parents could complete a simulator drive without experiencing simulator sickness given that their position in the car did not allow them to view the simulation from the correct eyepoint. The second visit included an acclimation drive and the study drive. The study drive was recorded using both audio and video for later transcription and coding. In addition to in-lab study procedures, all dyads received a dashboard camera at the end of the first study visit. Cameras were installed in their personal vehicle to record four supervised driving sessions, lasting between 10 and 30 min, occurring between the first and second study visit. These naturalistic driving recordings captured observational data of parent-teen communication during real world driving practice.

This report adheres to current SPIRIT guidelines [11] and was informed by the SPIRIT checklist (Supplementary Information). Additionally, the SPIRIT participant timeline outlines the schedule for enrollment, intervention activities, and assessments (Table 1).

**Table 1:** SPIRIT participant timeline

TIMEPOINT^b^	Enrollment		TRIAL PERIOD		
			Post-randomization		Close-out
	-t_i_ to 0	0	t_1_	t_2_	t_x_
**ENROLLMENT**:					
**Eligibility screen**	X				
**Informed consent**	X				
**Acclimation drive**		X			
**Randomization**		X			
**Dashcam installation**		X			
**INTERVENTION**					
***HazAPP***			X		
**ASSESSMENTS**:					
***Questionnaires***		X			
***Naturalistic drives – parent-teen communication***				X	
***Simulated study drive – parent-teen communication; teen driving performance***					X

#### Group 1 (Control)

Dyads in the control condition were not given access to the web-based program. Rather, parents and teens continued their driving practice at home as they normally would and were asked to record four driving sessions between visits using the provided dashcam. At the second study visit, the teen completed the standardized simulator drive that included common roadway hazards with the parent supervising from the passenger seat. Parents were given access to the program upon request after completing all study procedures.

#### Group 2 (Treatment)

At the conclusion of the first study visit, parents randomly assigned to the intervention condition were given a username and password that provided access to the HazAPP program. Parents completed the web-based program online, which introduced hazard anticipation concepts, demonstrated communication approaches, and provided examples across six common hazard categories (i.e., path conflict, stopping or slowing vehicles, roadside incursions, forced path change, obscured potential hazards, and emergency vehicle effects). The parent and teen then worked together on a guided hazard discussion activity. Like the control condition, dyads assigned to the intervention condition used a dashcam to record four supervised driving sessions between the two study visits. At the second study visit, dyads completed the same simulator drives as the control condition.

### Study population

The HazAPP RCT study enrolled 103 parent-teen dyads, of which 102 completed all study procedures. One dyad chose to withdraw from the study due to the parent experiencing motion sickness in the simulator at Visit 1. Teen age ranged from 14 to 17 years old, with 52 males and 50 females. Each teen participated with one parent or legal guardian. An even gender balance was achieved via sex-stratified randomization, given higher crash rates observed among male teen drivers (IIHS [4]), . Otherwise, participants were selected without regard to race, ethnicity, and other socioeconomic factors.

#### Eligibility criteria

The inclusion criteria were: (1) teen age 14–17, (2) holding only a valid instructional permit and no other type of license, and (3) the participating parent/legal guardian was the teen’s primary driving instructor. Exclusion criteria included: (1) teen being outside of the target age range, (2) parent not being the primary driving instructor, (3) teen not being in the instructional phase of licensure and/or holds a special minor’s restricted license, moped license, or intermediate license, and (4) the teen or parent reported a medical condition (e.g., Ménière’s disease) that would put them at increased risk of experiencing simulator sickness while engaging in the simulated driving task.

### Study procedures

#### Recruitment

Several methods were used to recruit parents and teens for study participation. These included mass emails to the university system, postings to social media platforms (e.g., Facebook), and the distribution of study flyers to surrounding school districts, driver education programs, churches, and businesses. Snowball sampling was also used.

#### Consent

At the start of the first study visit, parents and teens completed consent. An experimenter described all study procedures, risks of participation, and compensation. Parents and teens were encouraged to ask questions prior to consenting to study procedures. Note that only parents and teens who consented to take part in the study were randomized into one of the two study groups. Participants were given the choice to consent to storing their data in central repositories sponsored by the National Institutes of Health (NIH) or other federal agencies and sharing it with other qualified researchers. Participants were assured that their data would not be shared with anyone outside of the study team if they did not consent to it.

#### Randomization

Randomization was stratified by sex (male and female) to account for the substantially higher fatal crash rates observed among male teen drivers (e.g., IIHS [4]), , ensuring balanced representation across study arms and allowing sex to be included as a covariate in planned analyses. Prior to participant enrollment, two blocks of participant IDs were created. One block was for females, the other for males. Half of the IDs in each block indicated control condition assignment, while the remaining half indicated assignment to the intervention condition. These blocks were then entered into a list randomizer. Condition assignment strictly adhered to the randomly ordered list. Random assignment occurred after consent was complete.

#### Data collection

##### Visit 1

Upon arrival to their first visit at the Driving Safety Research Institute, both parents and teens completed an intake form verifying the teen’s licensure status and if the teen had been issued any driving citations that may impact study procedures. Additionally, we verified and recorded parent and teen licensure, as well as car insurance status. Following consent, the parent completed a set of questionnaires on Qualtrics (Provo, UT) using an iPad. While parents completed questionnaires, teens were taken to the simulator room. While in the simulator room, the teen watched a PowerPoint detailing the simulator operation (e.g., seat belt, seat adjustment, gear shift, etc.). Upon completion of the questionnaires, parents were brought to the simulator to begin the driving session with their teen. Teens and parents were situated in the driver’s and passenger’s seats, respectively, and asked to complete a wellness questionnaire reporting on symptoms related to simulator sickness. The initial survey established a baseline for later comparison. Following the wellness survey, parents and teens completed the acclimation drive.

The acclimation drive included downtown, residential, and rural driving environments similar in geography to the local city and surrounding area. It followed local speed limits ranging from 30 miles per hour (urban/suburban areas) to 45 miles per hour (rural areas). There were no hazards or traffic present during the acclimation drive. The purpose of the acclimation drive was to introduce parents and teens to the simulator and ensure they did not experience simulator sickness. The acclimation drive lasted approximately eight minutes.

At the conclusion of the acclimation drive, parents and teens completed the wellness questionnaire again. Scores on the post-drive wellness questionnaire were compared to baseline wellness to determine if simulator sickness was experienced by either dyad member. If either parent or teen score reached or exceeded the predefined threshold for nausea (21), oculomotor (32), disorientation/dizziness (15), or a total score of 32, participants were determined to be unable to complete the full study drive during the second study visit and were withdrawn from the study. In addition to completing the wellness survey, parents and teens were encouraged to let the experimenter know if they were experiencing any symptoms of simulator sickness during the drive. In these cases, the drive was stopped and the dyad was withdrawn from study procedures. Following the drive, teens completed a set of questionnaires in Qualtrics, while the parent was taken to another room and alerted to their condition assignment. Parents assigned to the intervention condition were given a brief introduction to HazAPP and told that they should complete the program within one week of the first study visit. Parents in both groups were shown how to use the dashcams for the naturalistic drives. Parents then scheduled their second study visit. Finally, parents and teens both completed a payment form.

##### Naturalistic drive capture

As a supplement to the simulator drives completed in Visit 2 and to aid in generalizability, naturalistic drives captured parent-teen conversation in an ecologically valid, real-world context. Both conditions were given and shown how to operate a DJI Action 2 camera. An experimenter installed the camera using a windshield mount. Parents were also shown how to install the mount in case they chose to change vehicles for supervised driving sessions. This system was used by parents to record four naturalistic drives they took, with their teen as the driver, between the two study visits. Parents were told that these drives should last between 10 and 30 min and should capture their usual driving lessons. Parents in the intervention condition were told to complete the web program prior to recording drives with their teen.

##### Visit 2

The second study visit took place approximately three to four weeks after the initial study visit. At this visit, dyads completed a hazard-laden simulator driving session to capture parent and teen interactions in response to roadway hazards. First, the parent was escorted to a quiet room, where they completed a baseline wellness survey while the teen rewatched the PowerPoint presentation from Visit 1 to refamiliarize themselves with the simulator. The teen completed the 8-minute practice drive alone to reacclimate to the simulator’s motion and responsiveness.

Parents joined their teens in the simulator cab after the teen completed the acclimation drive. As before, parents were in the front passenger seat. Prior to the start of the study drive, teens also completed the wellness survey. The teen and parent then completed the study drive, which lasted approximately 20 min. The study drive followed a similar route as the acclimation drive. The drive started in a downtown area, went through a residential neighborhood, followed by a rural area, and completed in a residential town. Unlike the acclimation drive, the study drive included a variety of typical roadway conditions and 17 potential hazard scenarios (Table 2). Note that no hazards evolved into critical driving events that would result in a crash. Full descriptions of each hazard event can be found in a previous report [12]. Some of the simulated hazards were direct reflections of hazards covered by HazAPP, whereas others represented novel hazards. Parents and teens were not given explicit instructions to communicate during the drive but were encouraged to treat it like an ordinary driving session. The experimenter was able to communicate with participants through a speaker installed in the cab as needed. Parent-teen verbal communication during the drive was captured using both audio and video for subsequent coding. At the conclusion of the drive, participants remained seated in the vehicle until the experimenter safely terminated the simulation. Before leaving the simulator, the parent and teen completed post-drive wellness questionnaires. After leaving the simulator, the parent and teen concluded the visit by returning the dashcam and completing payment forms.

**Table 2:** List of hazard scenarios included in study drive

Hazard Type	Traffic environment		
	Downtown	Residential	Rural
**Path Conflict**	Left lane impeded	Partial lane obstruction	
**Stopping Vehicles**	Platoon braking	Intermittent braking	Lead vehicle
**Roadside Incursions**	Parallel parked car	School zone Potential vehicle incursion	Deer
**Forced Path Change**	Work zone lane closure	Lane drop	
**Obscured Potential Hazards**	Obscured cross traffic Mid-block crosswalk	Obscured T-intersection 90% curve	
**Emergency Vehicles**	Ambulance	Fire station	

### Intervention components

#### The Hazard Anticipation Program for Parents (HazAPP)

HazAPP was developed to address the leading cause of young driver crashes: hazard anticipation errors. This web-based program provided parents with knowledge about the most common roadway hazards, the key principles that teens need to learn about each hazard type, and multiple examples of how parents might talk to teens in real time about hazards. It also culminated in a joint parent-teen session to reinforce learning and make parents aware of how their teen perceives roadway hazards. The conceptual framework for the program integrated guiding principles from sociocultural theory [13] and motivational interviewing [14]. Sociocultural theory suggests that children learn to regulate behaviors via shared social interactions with a more knowledgeable caregiver. Motivational interviewing techniques provide parents with a communication structure that strengthens teens’ involvement in the learning process.

The HazAPP program sought to improve teens’ hazard anticipation skills by providing parents with examples of how they might discuss hazards either before or after they are encountered on the roadway. The program was divided into three components. The first two, introduction and hazard exemplars, were completed by parents alone. The third, a joint hazard identification task, was completed by parents and teens together. The introduction highlighted teens’ lack of hazard anticipation skills and overviewed the motivational interviewing techniques that were integrated into HazAPP. The introduction also familiarized parents with six common hazard categories [15]. Each category was clearly defined, and parents were taught what teens need to understand about each category to safely navigate around them (Table 3).

**Table 3:**
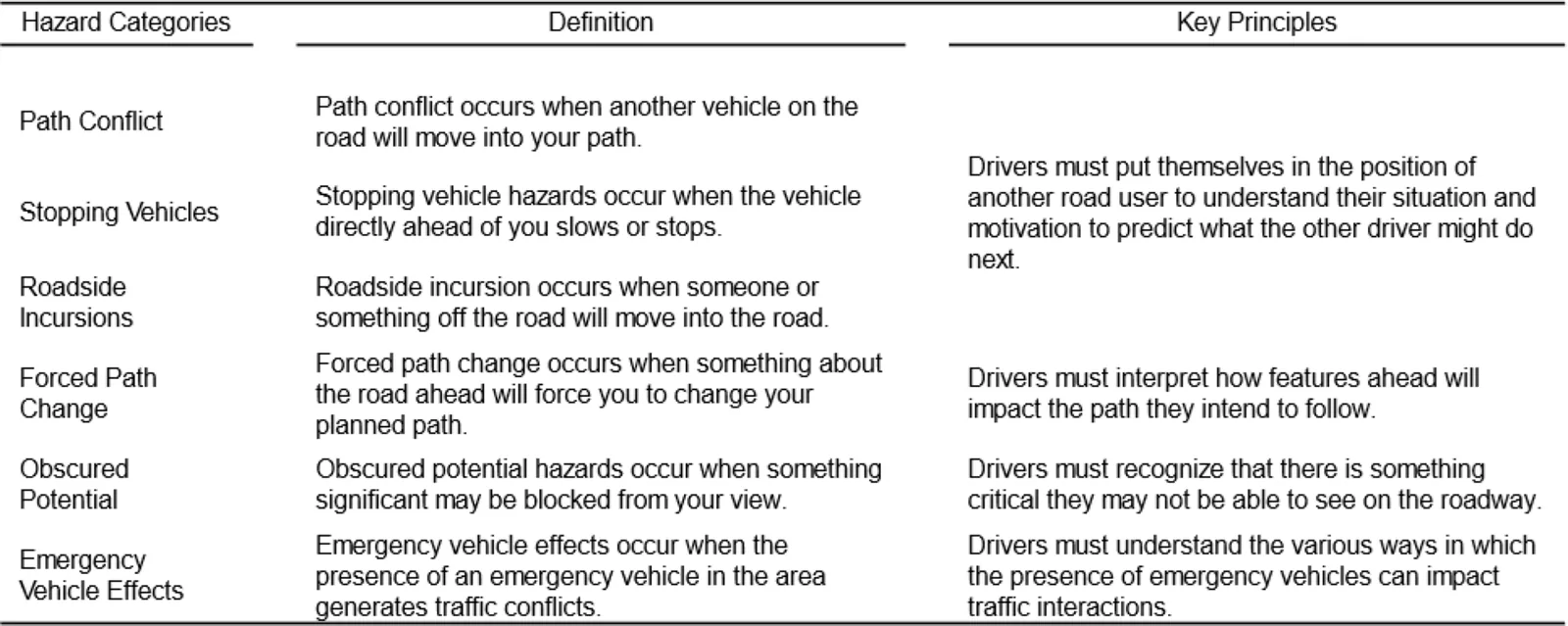
Hazard categories, their definitions, and key principles.

**Fig. 1:**
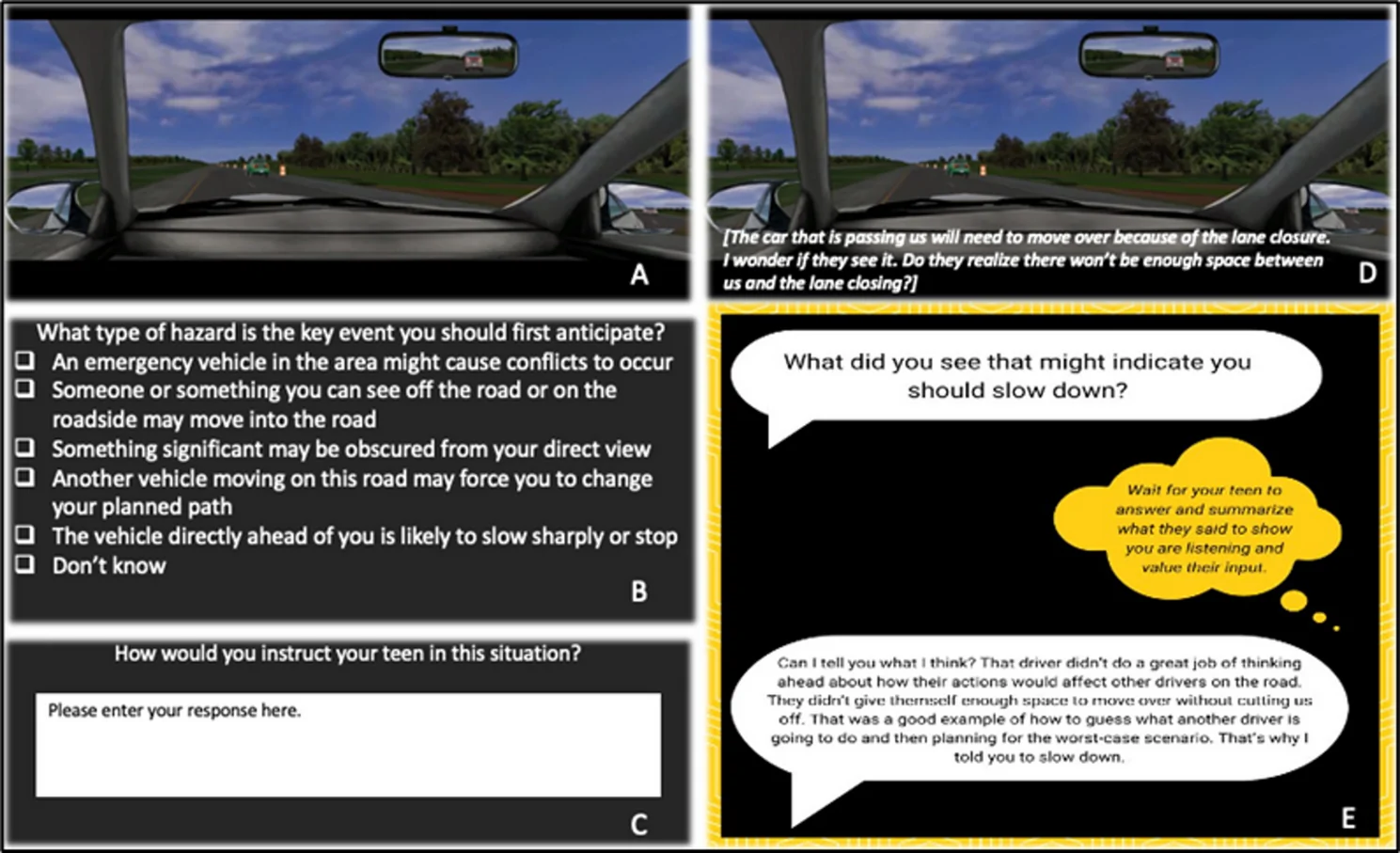
Example of a video scenario and the related tasks performed by parents as part of the intervention

Following the introduction, parents completed modules representing each of the six hazard categories. Each module contained six video exemplars. For each exemplar, parents first watched videos unfold (Fig. 1a) and were then asked to identify the key hazard event that should be anticipated using a multiple-choice list (Fig. 1b). Parents then indicated how they might talk to their teen about the hazard using a text input field (Fig. 1c). The video then replayed, accompanied by a voice over, demonstrating how parents can discuss each hazard before it becomes critical (Fig. 1d). Notably, these examples were drawn from parent-teen conversations captured during program development. This was followed by an example of how parents can discuss hazards after they have passed (Fig. 1e). After completing the training, parents and teens engaged in a joint session whereby parents and teens watched and discussed a set of 12 additional hazard scenarios (two from each hazard category). The joint task allowed parents to practice hazard communication without the added stress of navigating real roadway hazards. HazAPP could be accessed from internet connected devices with screens large enough to accurately view images and videos. The program took approximately 2 h to complete. However, the program did not have to be completed in a single sitting, allowing parents to complete the program at their own pace.

#### Intervention fidelity

To ensure completion of the HazAPP web program, researchers with administrative permissions regularly monitored program progress. Parents assigned to the intervention were sent a follow-up email 3 days after their initial appointment to remind them to complete the web-based program within a week before they recorded their naturalistic drives. After a week, if parents still had not made much progress on the web program, they were called to ensure that they hadn’t encountered any technical issues that impeded program completion and were encouraged to complete the program as soon as possible. Joint task completion was monitored via weekly reports. If their progress was still not completed before their next appointment, parents were offered the option to reschedule.

### Study measures

#### Primary outcomes

The primary study outcomes will be derived from the variables coded (Table 4) using recorded conversations that parents and teens had when engaging in the simulator and naturalistic drives.

Once reliable, study team members will code whether parents identify the hazard, whether the parent verbally identifies roadway hazards, if hazard identification uses a hazard anticipation communication (i.e., higher-order communication), if instruction precedes or follows the hazard event, if the hazard anticipation communication includes elements of motivational interviewing, and how many times parents provide functional driving instructions. Hazard anticipation communication, a form of higher-order driving instruction that can be applied more generally to driving (e.g., “When there are brake lights ahead, you should be ready to brake too.”), will be coded when parents indicate why the hazard is dangerous by causally connecting dangerous features and their potential outcomes. Functional instruction will be coded for instruction that applies only to the current context (e.g., “Slow down.”). We will also code whether the teen verbally identifies the hazard via hazard anticipation communication or by only pointing out the hazard and if identification precedes or follows the hazard event. Measures will be captured for each hazard that is presented in the simulator drive for a total of 17 repeated measures per dyad. For each of the 17 scenarios embedded in the simulator drive, a binary indicator reflecting presence or absence will coded for hazard anticipation communication, its sub codes, and hazard identification only. The number of functional driving instructions produced by the parent per hazard scenario will be recorded as a count.

Unlike simulator drives, hazards encountered in during naturalistic driving are not standardized. Therefore, coding will focus on general hazard anticipation patterns rather than per-hazard responses. Raw counts of each conversational measure will be recorded. because drives varied in length (10–30 min), counts will be modeled as a rate per 10 min of drive time. Each dyad contributed four naturalistic drives.

##### Coding procedures and reliability

Coding will follow an established coding scheme, informed by prior work on parent-child safety and parent-teen driving conversations (e.g [8, 16, 17]), . Two trained research assistants will code approximately 20% of transcripts to obtain interrater reliability. Coders will achieve substantial agreement as indicated by Cohen’s kappa statistic of 0.61 or higher for bivariate variables and an ICC of 0.75 or higher for continuous variables.

**Table 4:** Primary and secondary measures, operationalized, and coding scheme per data source

Outcome Variables	Measure/Operationalization	Simulator Drive Coding	Naturalistic Drive Coding
**Primary Outcomes**			
Hazard Anticipation Communication (HAC)	Higher-order communication that helps drivers identify and respond hazards by pointing out why they are critical and the clues on the roadway you can use to identify them. (e.g., “There could be something in front of the bus that we can’t see. They could pop out without warning.”)	Presence/Absence	Count
Before hazard	If HAC is present, does the HAC occur before or after the hazard would become critical if no action were taken	Presence/Absence	Count
Motivational interviewing	If HAC is present, are elements of motivational interviewing present in parent’s HAC?	Presence/Absence	Count
Functional Driving Instruction	Driving instruction related to vehicle handling and navigation (e.g., “Slow down.”, “Check your speed.”)	Count	Count
Hazard Identification Only	Identification of the hazard without any additional context or instruction (e.g., “I see a pedestrian.”, “That car is braking.”)	Presence/Absence	Count
**Secondary Outcomes**			
Risky Driving Events	Hard braking, speeding, hard accelerator press	Count	-
Lane Change	Occurs when the center of the participant vehicle crossed a lane boundary.	Presence/absence	-

#### Secondary outcomes

Secondary outcomes will focus on teens’ driving performance in the simulator. More specifically, simulator data will be reduced to capture risky driving events, changes to lane position, and lane changes in response to hazards and parental instruction. Risky driving events will include hard braking events, rapid acceleration, and speeding. Additionally, we will capture lane changes in response to hazards and parents’ instruction. Parental hazard anticipation communication is expected to prompt teens to adjust their speed and lane position proactively when approaching hazards, resulting in fewer hard braking events and more appropriate lane changes. These measures will be captured for each of the embedded hazards.

#### Other measures

Parents completed a set of questionnaires via Qualtrics during Visit 1, including demographics (i.e., race/ethnicity, educational background, and household income) and the Early Adolescent Temperament Questionnaire (Ellis [18]), . Additionally, questionnaires assessed parents’ driving safety beliefs, approach to and experiences with their teen while providing driving instruction, their typical communication style while supervising practice drives, and parents’ perspectives on their teen’s readiness to drive. These responses will be used to characterize the sample and to contextualize observed parent-teen communication during the simulator session. Teens also completed questionnaires via Qualtrics during Visit 1. These questionnaires included information about driving experience (i.e., permit date, driver education engagement, and frequency of recent driving exposure) and how often their parent discussed various driving topics during supervised practice. Teens also reported on their perceptions of their parent’s communication style using the Family Communication Pattern Scale – Revised [19] and the Parental Authority Questionnaire [20]. These measures will provide context for interpreting the in-drive communication recorded at Visit 2.

### Analytic plan

#### Data management

Participants were identified by number rather than by name. The master list linking participant IDs to participants identifiable information was saved on a password-protected data server accessible only to Institutional Review Board (IRB) approved members of the research team.

#### Questionnaire data

All questionnaire data collected from teens and parents are saved in a password-protected Qualtrics database. Study team members checked questionnaire data regularly for completeness. At the end of data collection, all questionnaire responses were downloaded and saved to a password-protected data server accessible only to IRB-approved members of the research team. These data were then cleaned and, where appropriate, coded for later analysis.

#### Simulator data

##### Video

Audio and video recordings of the hazard-laden simulator drives were transferred from the simulator hard drive to a password-protected data server accessible only to IRB-approved members of the research team. Files were checked once per week to ensure they were transferred without error. Researchers also made notes regarding any noteworthy events that occurred during the study drive. Audio from the video files will be used to generate verbatim transcripts for parent-teen conversations for later coding. Transcription will be conducted using University licensed AI, ensuring the protection of participant confidentiality.

##### Driving performance

Driving performance data gathered during the hazard-laden simulator drive were housed in a password-protected database managed by the Driving Safety Research Institute, home of the simulator used for data collection. Study team members ensured these data were properly transferred after each study visit. These data will be reduced for later analyses.

#### Naturalistic driving data

Audio and video recordings of the four naturalistic drives, captured by parents as they instructed their teens while driving on real roads, were downloaded from the camera’s memory card at the conclusion of the second study visit. Videos were checked for quality and completeness. In most cases, drives were separated into smaller video files by the DJI Action II camera. Study team members checked the videos and merged files chronologically to ensure that each of the four naturalistic drives were accessible as a single video file. Study team members also recorded noteworthy events and data quality issues (e.g., camera malfunctions). These videos will be transcribed verbatim in a similar manner as the simulator drives. Transcription of naturalistic video files will be conducted using Whisper AI and then imported to ELAN. Whisper AI can be run on a computer’s hard drive, ensuring participant privacy. Transcriptions will be coded in ELAN for later analysis.

#### Missing data

Missing data will be addressed to minimize bias and protect statistical power. To address missing data, we will begin by examining patterns of missingness. If rare, missing values will be assumed to be unrelated to study outcomes or relevant covariates and will be treated as if missing completely at random. We will proceed with analyses that ignore those observations. However, if missingness is deemed systematic or related to key variables, we will use a multiple-imputation approach, applying a simulation-based method grounded in a model that represents the missing data mechanism [21, 22].

#### Planned data analysis

##### Descriptive analysis

Data analysis will address our main hypothesis that parents who engage in the training will exhibit improved communication about potential hazard detection with their teens compared to those in the control condition. All analyses will include covariates of relevant demographic (e.g., sex, race/ethnicity) and driving related covariates (e.g., driver education engagement, duration of licensure, family communication patterns, etc.). P-value adjustments will be made as necessary.

##### Primary analysis

The primary set of analyses will examine the impact of the intervention on real-time parental instruction surrounding hazard identification and mitigation separately for the simulator and naturalistic drives.

**Simulator drives** Generalized linear mixed models will be used to predict conversational measures using a fixed effect of condition and covariates detailed above. For presence/absence measures (e.g., hazard anticipation communication), the probability of occurrence will be modeled using a mixed-effects logistic regression modeling approach. Condition will be included as a fixed effect, along with relevant demographic and driving covariates. Potential random effects of participant and hazard scenario will be determined using loglikelihood ratio testing. Outcomes will be interpreted as probability of occurrence. For count measures (e.g., functional driving instruction), the frequency of occurrence will be modeled using a mixed-effects position regression approach. Again, condition will be included as a fixed effect, along with relevant demographic and driving covariates and random effects structure determined with loglikelihood testing. Outcomes will be interpreted as incident rate ratios. Additionally, we will examine the distributions of teen temperament, family communication pattern, and parenting style and explore if intervention differences are suggested. P-value adjustments for multiple tests will be undertaken. We predicted that parents in the intervention arm of the study will exhibit increased probability and incidence of higher-order instruction of hazard anticipation and response with their teen.

**Naturalistic drives** The rate for each conversational outcome per 10 min of naturalistic driving will be modeled using a mixed-effects Poisson regression. The log of drive duration in 10-minute units will be included as an offset term to account for variation in drive length, such that model coefficients reflect expected counts per 10-miunte interval, rather than per drive. As with simulated driving, condition will be included as a fixed effect along with demographic and driving covariates. A random intercept of dyad will also be included to account for the correlation among the repeated drives within dyad. Drive number (1–4) will also be explored as a potential covariate to account for potential change over the naturalistic driving period.

##### Secondary analysis

The secondary set of analyses will examine any changes to teen’s driving behavior in response to parental instruction during the simulator drive. More specifically, we will use mixed-effects models to predict outcomes of risky driving behaviors and lane changes. Each model will include a fixed effect of condition and covariates detailed above. Potential random effects of participant and hazard scenario will be determined using loglikelihood ratio testing. P-value adjustments for multiple tests will be undertaken. We expected that teens of parents in the intervention arm of the study will exhibit improved driving responses to potential hazards in the simulator drive.

#### Sample size

Power calculations based on the medium to large effect sizes reported in previous studies of parent-focused intervention to improve teen driving^15,16^ indicated that a minimum of 100 dyads (*N =* 200 participants total) would be needed to detect medium-size effects at a 0.80 level of power. Power calculations were conducted in G*Power [23].

### Ethics

This study received ethical approval from the Institutional Review Board at the University of lowa [202010535]. Several measures were taken to ensure that individuals were not coerced into participation. First, during the recruitment processes, messages were added to all emails exclusively stating that individuals were not required to reply to emails and could choose to opt out from receiving them at any time. Second, during the consent process, participants were told that being involved in the study was completely voluntary and they could stop at any time. Participants were assured they would not lose any benefits to which they were otherwise owed had they chosen to cease participation in the study at any point. Participants were also encouraged to ask any clarifying questions during the consent process. Participant confidentiality was ensured during the consent process and maintained throughout the entire study timeline. Steps were taken to maintain participant confidentiality and compliance with all relevant rules and regulations: (1) all participant information (names, dates of birth, phone numbers, addresses) was kept on a digital master list that only the study team could access; (2) participant dyads were all assigned anonymous IDs that were used in place of their names on all study materials; (3) during the consent process, participants were informed of the risks and confidentiality concerns of being involved in research and were provided with details of storing and sharing their video and performance data. Participants were given the choice to consent to storing their data in central repositories sponsored by the National Institutes of Health or other federal agencies and sharing it with other qualified researchers. Participants were assured that their data would not be shared with anyone outside of the study team if they did not consent to it.

### Dissemination

We will use a variety of common methods to disseminate our study findings. These methods will include traditional academic outlets (e.g., peer reviewed journals, conference presentations, incorporation of findings into course content where appropriate) and key stakeholders (e.g., Iowa Department of Transportation, area driver educators, Teen Safe Driving Coalition, National Foundation for Teen Safe Driving). We will also disseminate our findings to study participants using lay language and infographics to aid comprehension. We will also work closely with University of lowa and Injury Prevention Research Center communication experts, who will disseminate our research findings to audiences via news and other outlets to ensure our findings have the greatest impact possible.

## Discussion

The Hazard Anticipation Program for Parents (HazAPP) was developed to improve teens’ hazard anticipation skills by providing parents with the needed tools to provide real-time hazard anticipation instruction to their teen in the learner phase of licensure. The current RCT seeks to evaluate the feasibility of a web-based program, HazAPP, and determine its ability to shift parents from reactive, functional instruction toward proactive hazard anticipation communication.

HazAPP is an innovative approach to improving teens’ hazard anticipation skill because it goes beyond existing systems of teen driver education to specifically train parents to teach teens in hazard anticipation – the leading cause of teen motor-vehicle crashes [5, 24, 25]. HazAPP also fills an important gap in teen crash prevention because there are currently no programs available for supporting parent instruction of roadway hazards. Additionally, HazAPP’s web-based design is highly accessible, making the program ideal for all families, including those who may lack access to other driving resources.

The current study design has several limitations. First, the sample size was determined using only medium-to-large effects sizes, making the detection of smaller, yet meaningful differences more challenging. However, repeated observations (i.e., simulated hazards) and planned analysis were both chosen to improve estimates. Second, the sample was recruited largely via convenience sampling in the University area. While efforts to broaden our recruitment pool using Facebook were beneficial for overall generalizability, more testing is needed to determine true generalizability. Third, teens were not given direct access to HazAPP, as the intention was to deliver the program to parents who are supervising teen driving. Future work should evaluate the impact direct access may have on subsequent teen driving performance. Fourth, while information regarding family communication patterns and parenting style were included, these may not adequately capture the quality of the parent-teen relationship. Fifth, the study captures a brief portion of the supervised driving period and does not quantify changes to teens’ independent driving behaviors or safety outcomes. Finally, while the study achieved an even sex balance and sex will be included as a covariate in all planned analyses, the current sample was not powered to detect sex-moderated intervention effects. Given that male teens face substantially greater crash risk, we will conduct exploratory analyses examining sex as a potential moderator of intervention effects. However, as these analyses were not preregistered, findings should be interpreted with appropriate caution and replicated in future work.

If effective, HazAPP will improve parental instruction when teaching teens to identify and respond to potential roadway hazards during the learner phase of licensure and may have important implications for crash prevention among novice teen drivers. Assuming our hypotheses are correct, next steps include conducting an efficacy trial with a longer follow-up period to better examine the impact of the intervention over the learner period of licensure. Additionally, future work should examine the impact of HazAPP on teens’ crash risk upon beginning to drive independently.

## Supplementary Information

Below is the link to the electronic supplementary material.

Supplementary Material 1

## Data Availability

The datasets used and/or analyzed during the current study are available from the corresponding author on reasonable request.

## Abbreviations

HazAPP: The Hazard Anticipation Program for Parents
NIH: National Institutes of Health
IRB: Institutional Review Board
RCT: Randomized Control Trial
